# Neonatal circumcision and prematurity are associated with sudden infant death syndrome (SIDS)

**Published:** 2019-01-09

**Authors:** Eran Elhaik

**Affiliations:** ^1^Department of Animal and Plant Sciences, University of Sheffield, UK

**Keywords:** sudden infant death syndrome (SIDS), unexplained mortality, allostatic load, neonatal circumcision, prematurity, trauma, pain, stress, lilith

## Abstract

**Background:**

Sudden infant death syndrome (SIDS) is the most common cause of postneonatal unexplained infant death. The allostatic load hypothesis posits that SIDS is the result of cumulative perinatal painful, stressful, or traumatic exposures that tax neonatal regulatory systems.

**Aims:**

To test the predictions of the allostatic load hypothesis we explored the relationships between SIDS and two common phenotypes, male neonatal circumcision (MNC) and prematurity.

**Methods:**

We collated latitudinal data from 15 countries and 40 US states sampled during 2009 and 2013. We used linear regression analyses and likelihood ratio tests to calculate the association between SIDS and the phenotypes.

**Results:**

SIDS mortality rate was significantly and positively correlated with MNC. Globally (weighted): Increase of 0.06 (95% CI: 0.01-0.1, *t* = 2.86, *p* = 0.01) per 1000 SIDS mortality per 10% increase in circumcision rate. US (weighted): Increase of 0.1 (95% CI: 0.03-0.16, *t* = 2.81, *p* = 0.01) per 1000 unexplained mortality per 10% increase in circumcision rate. US states in which Medicaid covers MNC had significantly higher MNC rates (*χ̄* = 0.72 vs 0.49, *p* = 0.007) and male/female ratio of SIDS deaths (*χ̄* = 1.48 vs 1.125, *p* = 0.015) than other US states. Prematurity was also significantly and positively correlated with MNC. Globally: Increase of 0.5 (weighted: 95% CI: 0.02-0.086, *t* = 3.37, *p* = 0.004) per 1000 SIDS mortality per 10% increase in the prematurity rates. US: Increase of 1.9 (weighted: 95% CI: 0.06-0.32, *t* = 3.13, *p* = 0.004) per 1000 unexplained mortalities per 10% increase in the prematurity rates. Combined, the phenotypes increased the likelihood of SIDS.

**Conclusions:**

Epidemiological analyses are useful to generate hypotheses but cannot provide strong evidence of causality. Biological plausibility is provided by a growing body of experimental and clinical evidence linking aversive preterm and early-life SIDS events. Together with historical and anthropological evidence, our findings emphasize the necessity of cohort studies that consider these phenotypes with the aim of improving the identification of at-risk infants and reducing infant mortality.

**Relevance for patients:**

Preterm birth and neonatal circumcision are associated with a greater risk of SIDS, and efforts should be focused on reducing their rates.

## 1. Introduction

Sudden Infant Death Syndrome (SIDS) occurs when a seem-ingly healthy infant (0-12 months) dies unexpectedly in sleep with no cause of death established in a post-mortem investiga-tion [1]. SIDS is the leading cause of infant death in many de-veloped countries [2], accounting for 2,700 deaths annually in the US [3]. As such, SIDS has received much attention in the literature (e.g. [4]).

The allostatic load hypothesis for SIDS [5] purports that pro-longed and repetitive exposure to stressors (e.g., poor postnatal weight gain [6], hyperthermia [7], and maternal smoking [8]) is maladaptive and has a cumulative effect that increases the risk of SIDS. It differs from the triple risk hypothesis [5], which posits that SIDS is caused by an exposure to intrinsic and external fac-tors during a critical developmental stage. That hypothesis can-not explain the four main characteristics of SIDS, namely male predominance (60:40) [9], the 39% lower SIDS rate among US Hispanic compared to non-Hispanic people [10], the seasonal variation with most cases occurring in winter [11], and that 50% of cases occur between 7.6 and 17.6 weeks after birth with only 10% past 24.7 weeks.

To test the predictions of the allostatic load hypothesis for SIDS, we identified two common phenotypes [5], male neonatal circumcision (MNC) and premature birth, for which latitudinal data were available and tested their association with SIDS. Both phenotypes are male-biased [12] and may explain the male pre-dominance of SIDS, whereas the first phenotype may also ex-plain the lower SIDS rate in Hispanic people.

MNC is associated with intraoperative and postoperative complications including bleeding, inadequate skin removal, sur-gical site infection [13], inflammation and sepsis, circulatory shock, traumatic injury that result in partial or complete pe-nile amputation or other injury to the penis [14], chordee, iatro-genic hypospadias, glanular necrosis, glanular amputation [13], and hemorrhage [15–18] that can result in death [17,19]. MNC can cause clinically significant pain despite the use of analgesia and severe pain when no analgesia is used [20–22]. The proce-dure has been associated with “strikingly significant changes in physiological, hormonal and behavioral parameters, and adverse events such as choking and apnea” [23], both precede sudden death. Several recent longitudinal studies attempted to assess the short-and long-term complication rates of MNC. For example, a five-year long California study of 24,432 circumcised children under age 5 reported cumulative complication rates of 1.5% in 0-3 months neonates (0.5% of which are within the first 30 days of life) and 2.9% in 3 months-5 years old non-neonates (2.2% of which were early) [18]. A two-year long study in Canada of 277 patients (mean age at recruitment 28.4 days) reported com-plication in 12.6% of the patients, of which 6.5% experienced excessive bleeding and 9.4% long-term complications [24]. A two-year long study in Utah of 6,298 neonatal circumcisions found a complication rates of 11.5% with 1.6% of the patients undergoing surgical revision or lysis of penile adhesions [25]. A two-year long New York study of 1,064 neonatal circumcisions reported complications in 3.9% of the patients [26]. Common expressions of extreme distress in response to circumcision in-clude “very strained and labored upper limb movements, high-pitched screeches, bilateral arm raising and widening, breath holding, abrupt and intentional arm movements, and frantic up-per limb movements” [27]. Pain during wound-healing for new-born circumcision has been observed up to 6 weeks following the surgery [28], as the exposed glans may come into contact with urine and feces. MNC involves maternal separation and re-straint to a board with removal of highly sensitive penile tissues, which may increase the risk of long-term adverse psychosexual sequelae [29–32]. Research suggests that procedures that are far milder than MNC, such as routine heel punctures, can have persistent negative effects with changes to immune, endocrine, and behavioral reactions to stressful events continuing into child-hood or even adulthood [33,34]

Deaths following MNC have been known for a long time as also acknowledged in the Talmud [35] to present time [36] with most deaths associated with excessive bleeding, infection, and less frequently with anesthesia accidents and cardiac arrest (re-viewed in [37]). Recently, Earp et al. analyzed data from a US inpatient database of nearly 10 million MNCs over 10 years. The authors attributed an early death rate of 2/100,000 to MNC. The risk of early death (first 30 days) increased for infants circum-cised in a teaching hospital and if comorbid conditions (e.g., car-diac diseases) are present [36]. This death rate should be consid-ered an underestimate, provided the lack of systematic collection of mortality statistics associated with non-therapeutic circumci-sion in the US, which precludes, for instance, tracking deaths occurring in a hospital other than the hospital where circumci-sion took place.

Since MNC preference is largely cultural, populations can be classified into Anglophone countries (high MNC rate) and non-Anglophone countries (medium to low MNC rate [38,39]) (Ta-ble S1). If MNC is a risk factor for SIDS, SIDS rates would be higher in Anglophone countries, where MNC is highly prevalent [38], compared to non-Anglophone countries, which tradition-ally have opposed circumcision [39]. In the US, male circumci-sion is usually done in the neonatal period, but US populations differ in their MNC practices. A comparison of the circumcision rates among males (14-59) between 2005 and 2010 found that non-Hispanic Whites (NHW) were the group with the highest circumcision rates (90.8%), followed by non-Hispanic Blacks (NHB) (75.7%) and Mexican Americans (44%) [40]. If MNC is a risk factor for SIDS, in addition to their low SIDS rates we can also expect Hispanic populations to exhibit lower male bias in unexplained deaths than non-Hispanic.

Prematurity (birth at a gestational age of less than 37 weeks) is a known risk factor for SIDS [41,42]. The risk factors unique to preterm infants likely have multiple etiologies and include biological vulnerabilities and prolonged exposure to multiple stressors during the hospitalization in the neonatal intensive care unit (NICU) [41], which elevates the allostatic load and the risk for SIDS [43].

We tested the association of SIDS with these two phenotypes using data from 16 worldwide populations, 15 countries, and 40 US states. This is the first study to test the association between SIDS and MNC, and it is done at a time that SIDS rates remain relatively high [44] two decades after the “Back to Sleep” (BTS) campaign.

## 2. Methods

### 2.1. Data collection

*The global dataset*. SIDS rates (Table S1). We collected SIDS data (2004-2013) for 15 countries [10,45,46]. The SIDS rate is calculated as the number of deaths per 1000 live births. *Male neonatal circumcision (MNC)*. MNC rates per country (2005-2013) were obtained by searching for ‘neonatal circum-cision’ and country in Google Scholar, Google, and PubMed. Similarly to the method employed by Bauer and Kriebel [47], MNC rates for the remaining countries that could not be obtained through peer reviewed journals and whose adult circumcision rates were estimated by the WHO to be <20% [38] from the to-tal percentage of Muslims [48] and Jews [49] in the country, as both populations were reported to have 100% circumcision rate [50]. Unlike Jewish traditions where ritualistic circumcision is performed on the eighth day after birth, Islamic traditions do not provide a specific recommendation and the age of circumcision varies according to family, parents’ education, Islamic branch, country of origin [51], MNC costs, and the contemporary coun-try’s norms and legislation [52]. Nonetheless, a sizeable pro-portion of circumcisions are done neonatally in Iraq [53] (18% were circumcised in the first 180 days), Norway [54] (20% were circumcised in their first year), Pakistan [55] (44% were circum-cised in the first 60 days), and Turkey [56,57] (14.8% were cir-cumcised in their first year, half of them within the first 30 days). In Belgium, between 1994 and 2012, the age of which a child is circumcised has decreased [58]. These variations will have min-imal effect on our analyses, provided the average low MNC rates in the countries where they were estimated from the Muslim and Jewish populations. *Prematurity rates*. Prematurity data (2004-2013) were obtained from the March of Dimes Foundation [59].

*The US dataset*. Unexplained mortality rates (Tables S2, S3). Mortality records were obtained from the Centers for Disease Control and Prevention (CDC) Wonder [10] database “Com-pressed Mortality (1999-2016)” for infants (<1-year-old). The database “Compressed Mortality (1979-1998)” for infants (<1-year-old) was used in Figure 10. Due to the limited amount of data on SIDS (R95), we used the ICD10 codes for all ill-defined and unknown causes of mortality (R95-R99). The un-explained mortality rate is calculated as the number of deaths per 1000 live births. The gender bias was calculated as 1000 *− M_SIDSrate_*/*F_SIDSrate_*. *MNC rates*. US statewise for male newborn births and MNC rates for 2013 were obtained from the US Department of Health Human Services (HCUP) [60] us-ing ICD-9-CM diagnosis codes V30-V39 and ICD-9-CM proce-dure code 64.0 (“Circumcision”). Data for the remaining states were obtained from the 2009 data in the Kids’ Inpatient Database (KID), Healthcare Cost and Utilization Project (HCUP), Agency for Healthcare Research and Quality [61]. 2009 was the last year when KID recorded state information. Statewise Medicaid cov-erage for MNC was obtained from [62] for all states and [63] for Indiana. In calculating the SIDS gender bias for Hispanic and non-Hispanic populations (Table S3) using the CDC Wonder data [10], we analyzed states where the population of Hispanic exceeded 12.5% (the average number of Hispanic people in the US). *Prematurity rates (Table S2)*. Best year-match US statewise prematurity data were obtained from [64]. *Census data (Table S3)*. Data were obtained from the US Census (2000, 2010) [65] and the 2012-2016 American Community Survey 5-Year Esti-mates [66].

**Figure 1:**
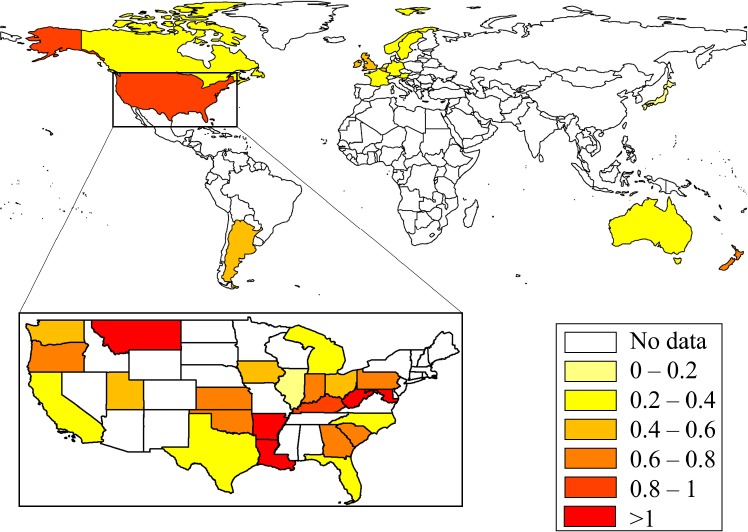
Male SIDS mortality rates (per 1000 births) in 15 countries and 40 US states (inset). SIDS mortality rates are color-coded.

**Figure 2:**
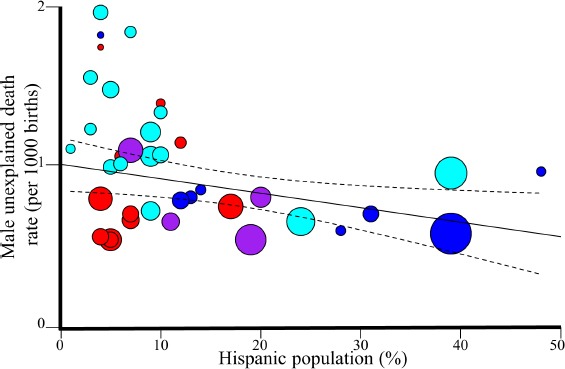
Regression analysis of Hispanic in the US and unexplained male mortality rates in 2015. The 95% confidence intervals of the best fit line are denoted in dashed lines. Colors correspond to four US regions: Northeast (violet), Midwest (red), South (cyan), and West (blue).

**Figure 3:**
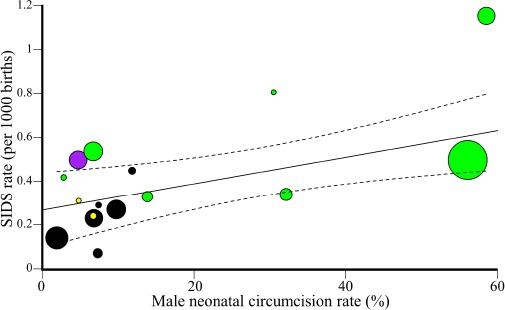
Weighted regression analysis of male SIDS mortality and global MNC rates. The 95% confidence intervals of the best fit line are denoted in dashed lines. Colors correspond to the four population groups: Anglophone countries (green), Ibero-American countries (violet), Nordic countries (yel-low), and all other (black). Circle size represent the relative population size.

**Figure 4:**
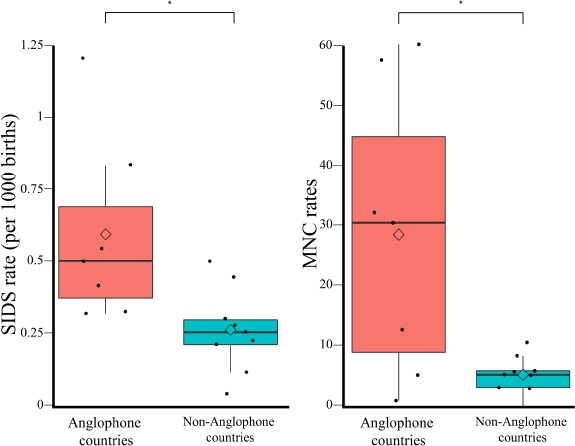
A comparison of the male SIDS mortality (left) and MNC (right) rates in 7 Anglophone and 9 non-Anglophone countries using boxplots. Sig-nificance was assessed with two-tailed t-tests.

**Figure 5:**
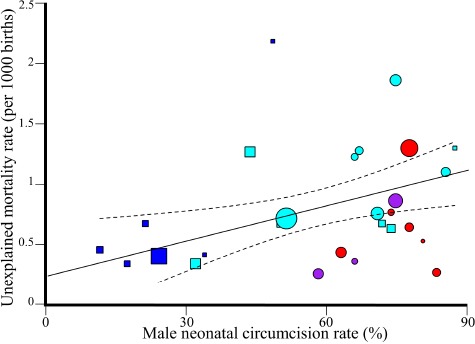
Weighted regression analysis of unexplained male mortality and US MNC rates. The 95% confidence intervals of the best fit line are denoted in dashed lines. Color codes are as in Figure 2. Symbols mark states where Medicaid, the leading insurance company in US, covers (circles) or does not cover (squars) MNC.

**Figure 6:**
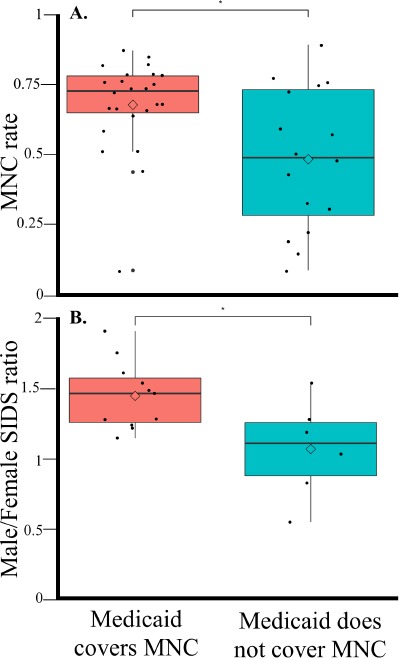
MNC rates and SIDS gender bias in US states as a function of Medicaid coverage of MNC. A comparison of MNC rates (A) and SIDS gender bias (B) in US states where Medicaid does or does not cover MNC using boxplots. Diamonds show the mean. Significance was assessed with two-tailed t-tests.

**Figure 7:**
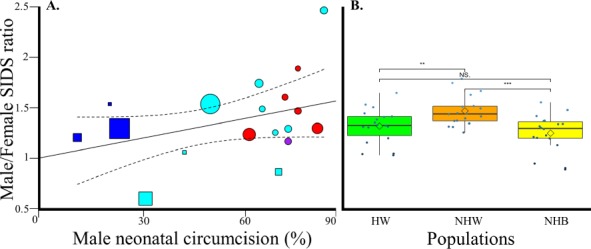
The contribution of MNC toward SIDS gender bias in the US. A) Weighted regression analysis of gender bias and US MNC rates. The 95% confidence intervals of the best fit line are denoted in dashed lines. Color codes and symbols are as in Figure 2. B) A comparison of the gender bias in three US populations using boxplots. Diamonds show the mean. Significance was assessed with two-tailed t-tests.

**Figure 8:**
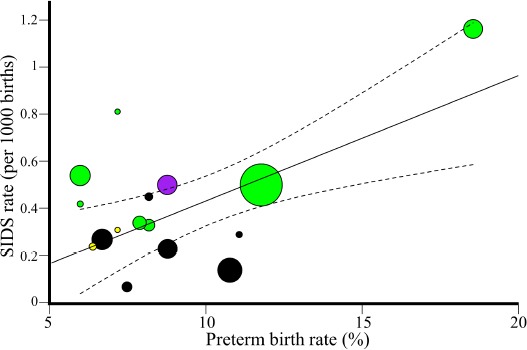
Weighted regression analysis of global male SIDS mortality and prematurity rates. Data were obtained for 15 states and 16 populations. The 95% confidence intervals of the best fit line are denoted in dashed lines. Color codes are as in Figure 3.

**Figure 9:**
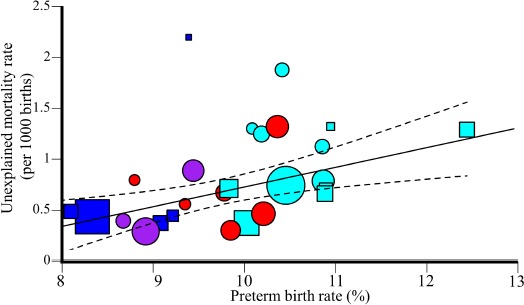
Weighted regression analysis of unexplained male mortality and prematurity rates in US states. The 95% confidence intervals of the best fit line are denoted in dashed lines. Color codes and symbols are as in Figure 2.

**Figure 10:**
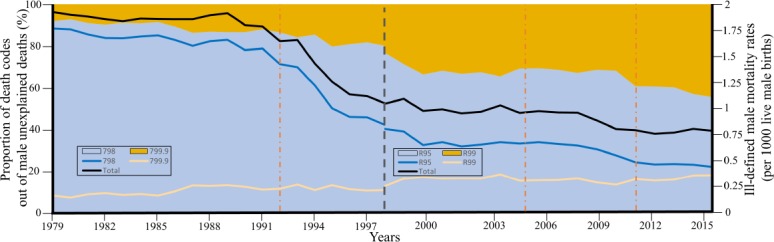
Trends in unexplained male mortality in all US states between 1979 and 2016 [10]. Data were obtained for SIDS all other ill-defined death codes, which represent 99% of unexplained death classification according to ICD 9 (left) and 10 (right). The grey bar represent data used by either ICD classifi-cation. Orange bar represent years in which the AAP recommended the supine position. In 1992 the AAP discouraged putting infants to sleep prone [155]. In 2005, the supine position was recommended exclusively [156], a recommendation which was confirmed in 2011 [157]. Areas show the percent of death classification codes to SIDS (798 or R95) or other ill-defined and unspecified causes of mortality (799.9 or R99). Lines show the rates of all unexplained mortalities according to each code and the total.

### 2.2. Data analyses

The global SIDS mortality rate map was plotted with R packages rworldmap [67] (V1.3-6) and maptools [68] (V0.9-4). All correlations were calculated using Pearson correlation using the R packages ggplot2 [69] (V3.1.0) and ggsignif [70] (V0.4.0). Linear regression analyses performed using ‘lm’ func-tion. Mixed effects model were calculated using the packages ‘lme4’ [71] (V1.1-19) and ‘lmerTest’ [72] (V3.0-1). Likeli-hood ratio tests were performed using the R package ‘lmtest’ [73] (V0.9-36). Analyses were done in R v.3.5.1. All data and code used in our analyses are available at GitHub (https://github.com/eelhaik/SIDS_study).

## 3. Results

### 3.1. Mortality rate

SIDS mortality rates varied greatly among the studied coun-tries, ranging from 0.06 to 0.82 per 1000 births ( *χ̄* = 0.4, *σ* = 0.27) (Figure 1, Table S1). SIDS mortality rates were the lowest in the Netherlands (0.06) and highest in the US (0.82) and New Zealand (0.8). The average SIDS mortality rate in the US was notably high compared with Europe (*χ̄* = 0.3, *σ* = 0.14). In the US, New York had the lowest unexplained mortality rate (0.31) and Montana the highest (2.16).

Considering the proportion of US Hispanic (12.5%) in the 2000 US census as a cutoff and weighting by the Hispanic pop-ulation size, the unexplained mortality rate was significantly lower in US states with high Hispanic population (h*H*) than states with low Hispanic population (l*H*) every year between 2000 and 2015 (Welch two-tailed *t*-test 2000: *N_hH_* = 8, *N_lH_* = 29, ∆*_mortality_*(*lH, hH*) = 0.32, *t* = 2.83, 95% CI: 0.09-0.55, *p* = 0.008, 2010: *N_hH_* = 10, *N_lH_* = 27, ∆*_mortality_*(*lH, hH*) = 0.53, *t* = 4.43, 95% CI: 0.29-0.78,*p* = 8.7 − 10*^−^*5; 2012: *N_hH_* = 11, *N_lH_* = 28, ∆*_mortality_*(*lH, hH*) = 0.44,*t* = 3.65, 95% CI: 0.2-0.69,*p* = 9 − 10*^−^*4; 2015: *N_hH_* = 11, *N_lH_* = 27, ∆*_mortality_*(*lH, hH*) = 0.39, *t* = 4, 95% CI: 0.19-0.58, *p* = 3 − 10*^−^*5). In other words, assuming an average unexpected mortality of 100 males per 100,000 births, states with a higher than average population of Hispanic residents will experience 40 fewer male unexplained deaths. Assuming a mixed effect model, where Hispanic origins and year were the fixed effects and state as the random effect, we found that Hispanic origins has a significant effect (two-sided *t*-test, *t* = −2.6, *p* = 0.01). The unexplained mortality rate in males was also significantly negatively correlated with the percent of Hispanic people in the population each year (Weighted two-tailed t-test 2000: *N* = 37, *r* = −0.25, β = −0.8, 95% CI: −1.62–0.01, *p* = 0.05; 2010: *N* = 37, *r* = −0.4, β = −1.2, 95% CI: −2.21–0.21, *p* = 0.02; 2012: *N* = 39, *r* = −0.34, β = −0.98, 95% CI: −1.89–−0.06, *p* = 0.04; 2015: *N* = 38, *r* = −0.36, β = −0.96, 95% CI: −1.76–0.15, *p* = 0.02) (Figure 2, Table S4).

### 3.2. MNC is positively associated with the risk for early mortality

The global SIDS and MNC rates are significantly correlated (Unweighted: *N* = 16, *r* = 0.7, β = 0.01, 95% CI: 0.004-0.015, *t*-test, *t* = 4,*p* = 0.003; Weighted: *N* = 16, *r* = 0.7, β = 0.0057, 95% CI: 0.001-0.01, *t*-test, *t* = 2.83,*p* = 0.012) (Figure 3). The results remain significant even if the MNC rates for the estimated cohort are halved or doubled (in both cases: Unweighted: *r* = 0.69 − 0.7, *p* = 0.003; Weighted: *r* = 0.69-0.7, *p* = 0.01). When dropping two random points and repeating the analysis 1000 times, the *p*-value was significant (*p* < 0.05) 88% of the time and the mean β was 0.01.

The slope of this trend indicates that a 10% increase in the MNC rates is associated with an increase of 0.1 per 1000 SIDS cases (*F* = 8.19, *p* = 0.01). Anglophone countries practice sig-nificantly more MNC and have significantly higher SIDS mor-tality rates than non-Anglophones (two-tailed *t*-test assuming unequal variance, *p* = 0.04 and *p* = 0.03, respectively) (Figure 4).

The US state-wise unexplained mortality and MNC rates are significantly correlated (Unweighted: *N* = 27, *r* = 0.28, β = 0.006, 95% CI: −0.002-0.013, *t*-test, *t* = 2,*p* = 0.15; Weighted: *N* = 27, *r* = 0.28, β = 0.009, 95% CI: 0.002-0.016, *t*-test, *t* = 2, *p* = 0.01) (Figure 5, Table S2). Similarly to the global trend, the slope of this trend indicates that a 10% increase in the MNC rates is associated with an increase of 0.09 per 1000 SIDS cases (*F* = 7.55, *p* = 0.01).

Male predominance is one of the hallmarks of SIDS. In 21 out of 40 US states where Medicaid, the most common US health insurance, covers MNC (Table S2), the average MNC rate is nearly 1.5 fold higher than the MNC rate in other states (*χ̄* = 72% vs 49%, Welch two-sided *t*-test, *t* = 2.7, *p* = 0.01) (Figure 6A), in agreement with Leibowitz et al. [74] (69.6% and 31.2%, respectively). The unexplained mortality rate is higher (*χ̄* = 0.79 vs 0.69, Welch two-sided *t*-test, *t* = 0.21, *p* = 0.82), although not statistically significant, and the SIDS male gender bias is significantly higher (*χ̄* = 1.48 vs 1.125, Welch two-sided *t*-test, *t* = 2.6, *p* = 0.02)(Figure 6B).

In US states, there is a high positive correlation between the MNC rate and SIDS gender ratio (Unweighted: *N* = 18, *r* = 0.38, β = 0.67, 95% CI: −0.18-1.52, *t*-test, *t* = 1.66, *p* = 0.11; Weighted: *N* = 18, *r* = 0.38, β = 0.63, 95% CI: −0.13-1.4, *t*-test, *t* = 1.74, *p* = 0.1) (Figure 7A). It is likely that the results were insignificant due to insufficient data, however the *r*2 inferred in the regression analysis suggests that MNC may explain 16% of the variability in male SIDS deaths in the US. Grouping the results by population, US states with a high population of Hispanic Whites (*>* 12.5%) had significantly lower SIDS gender bias compared to NHW (Welch two-sided *t*-test, *t* = −2.78, *p* = 0.008), which also have the highest MNC rates. NHB, who have intermediate MNC rates, also show lower SIDS gender bias compared to NHW (Welch two-sided *t*-test, *t* = −2.64, *p* = 0.0002) between 1999 and 2016 (Figure 7B, Table S3).

### 3.3. Prematurity is positively associated with the risk for early mortality

To test the association between prematurity and SIDS, we considered the global and US prematurity rates. Prematurity rates (%) are the highest in the US (18.46% and 11.7% for NHB and NHW, respectively) and lowest in Nordic countries (7%) (Table S1). The global SIDS mortality and prematurity rates are significantly correlated (Unweighted: *N* = 16, *r* = 0.57, β = 0.05, 95% CI: 0.008-0.08, *t*-test, *t* = 2.6, *p* = 0.02; Weighted: *N* = 16, *r* = 0.57, β = 0.05, 95% CI: 0.02-0.086, *t*-test, *t* = 3.37, *p* = 0.004) (Figure 8). The slope of this trend indicates that a 10% increase in the prematurity rate is associ-ated with an increase of 0.5 per 1000 SIDS cases (*F* = 11.37, *p* = 0.004).

US states also exhibit a significantly positive correlation be-tween unexplained mortality and prematurity rates (Unweighted: *N* = 27, *r* = 0.39, β = 0.18, 95% CI: 0.006-0.35, *t*-test, *t* = 2.13, *p* = 0.04; Weighted: *N* = 27, *r* = 0.39, β = 0.19, 95% CI: 0.06-0.32, *t*-test, *t* = 3.13, *p* = 0.004) (Figure 9). An increase of 10% in preterm rate is associated with an increase of 1.8 per 1000 unexplained mortality cases (*F* = 9.8, *p* = 0.004).

Due to the known male bias in preterm births [12], we tested whether prematurity rates explain the SIDS gender bias in US states. We found insignificant correlation between the prema-turity rate and SIDS gender ratio (*N* = 18; *r* = −0.06, β = −0.02, 95% CI: −0.21-0.17, *t*-test, *t* = −0.23, *p* = 0.8). In the US (*N* = 40, *r* = 0.33, β = 0.07, 95% CI: 0.006-0.14, *t*-test, *t* = 2.2, *p* = 0.03) and global datasets (*N* = 16; *r* = 0.67, β = 4.14, 95% CI: 1.56-6.72, *t*-test, *t* = 3.45, *p* = 0.0039), MNC and prematurity were significantly correlated, suggesting a potential confounder effect.

### 3.4. Additive effects of various phenotypes increase the risk of early mortality

A weighted multivariable model of US unexplained mortal-ity that includes MNC, prematurity, and region of the country found that MNC (β = 0.013, 95% CI: 0.004-0.02, *t* = 3.05, *p* = 0.006) and geographic region (*F* = 4.65, *p* = 0.005) were significant factors, while prematurity was not one (β = 0.02, 95% CI: −0.26-0.3, *t* = 0.17, *p* = 0.87).

To assess the additive effect of MNC and prematurity, we performed likelihood ratio (LR) tests considering all possible combinations of the phenotypes. We found in the global dataset that the combination of MNC and prematurity is a significantly better predictor of SIDS compared to MNC or prematurity alone (LR test, *p_MNC_* = 0.002, *p_Preterm_* = 5.32 − 10*^−^*5). In the US dataset, the combination of MNC and prematurity is a sig-nificantly better predictor of unexplained mortality compared to MNC alone but not prematurity (LR test, *p_MNC_* = 0.046, *pPreterm* = 0.4).

## 4. Discussion

Sudden infant death syndrome (SIDS) is a complex, mul-tifactorial disorder. In spite of continuous research and global Back To Sleep (BTS) campaigns, SIDS remains one of the most common and poorly understood diagnoses of death among in-fants between birth and 1 year of age [3,75]. Although SIDS affects infants from all social strata, NHB and NHW infants of lower socioeconomic status are at higher risk [75], whereas His-panic infants, albeit from lower socioeconomic statuts, paradox-ically do not demonstrate this link [76]. SIDS is also male pre-dominant. We speculated that MNC can explain these two cor-relations. We found that Anglophone countries practice signif-icantly more MNC and have significantly higher SIDS mortal-ity rates than non-Anglophones. Similarly, we found that US states where Medicaid covers MNC have significantly higher MNC, unexplained mortality rates, and SIDS male bias than ther states. Not only do infants of Hispanic origin suffer less from SIDS, they also have significantly lower SIDS male bias than NHW and NHB. MNC can explain 16% of the variabil-ity in male SIDS deaths in the US (*p* = 0.1). We further found that there is a strong and significant correlation between the rates of both prematurity and MNC and SIDS using global and US datasets. In the global dataset the two phenotypes predict SIDS better than each phenotype separately, whereas in the US MNC and prematurity predict unexplained mortality only better than MNC.

Much of the difficulties in studying SIDS pertains to termi-nological [77] and methodological problems [78]. SIDS is a di-agnosis of exclusion given when the cause of death cannot be determined. Therefore, SIDS can be expected to decrease over time as parental education and diagnostic methods improve. In-deed, the rate of SIDS has been declining worldwide since the 1980s [79] and has been accommodated by an increase in the mortality rate of sudden and unexplained infant deaths (SUIDs) -a diagnosis used to describe the sudden and unexplained death of a baby less than 1 year old in which the cause of death was not obvious before an investigation [80]. Interestingly, much of the decline in SIDS rates following the BTS campaign has been due to an increase in SUID deaths and other death classifica-tions [81], attesting to the limited success of the BTS campaign in preventing unexplained deaths [79]. Though SIDS mortality rate decreases with time as more causes of deaths are becom-ing known, it may also decrease due to the variability in, and confusion about, categorizing deaths [82] or inconsistency be-tween investigators [83]. The causes of death may also inten-tionally be misrepresented in order to avoid an autopsy due to cultural or religious practices or to avoid time-consuming in-vestigations [46]. Ontario, for example, eliminated all SIDS-related deaths between 2014 and 2016 by re-categorizing them as “undetermined” deaths [78]. In Kansas, only 4.7% of all un-explained deaths between 1999 and 2012 were classified as ill-defined mortalities (R99), but by 2016, they represented 37% all unexplained deaths, reflecting a decrease of 32.5% in the share of SIDS deaths. The actual decline in unexplained mortalities (R95+R99) in the US during these periods was much modest (10%) [10]. Considering all US states (Figure 10), between 1979 and 1998, unexplained deaths decreased by 54%, but SIDS de-clined by 46%, whereas other ill-defined deaths (R99) climbed by 33%. Between 1999 and 2016, unexplained deaths decreased by 25%, but SIDS declined by 46%, whereas other ill-defined deaths (R99) climbed by 44%. SIDS represented 92% of all un-explained deaths in 1979 and 55% by 2016. These trends demon-strate the challenges of using longitudinal data to study SIDS and imply that the interest in studying the contemporary SIDS rates conflates with the amount of available SIDS data.

Daunting methodological problems are also prevalent in SIDS studies. The unavailability of proper controls and inabil-ity to account for the different life histories of infants beginning *in utero* and their exposure to environmental stressors later in life (e.g. [84]) are major limitations in SIDS studies. Cohort studies are also problematic due to the difficulty of finding suit-able controls and accounting for external stressors, which vary widely among countries, cultures, and socioeconomic statuses and can render association studies ambiguous. These method-ological difficulties have resulted in over 100 explanations for SIDS that appeared in *Medical Hypotheses* [5] and much confu-sion between cause and effect. For instance, it has been reported that breastfeeding for a duration of at least two months is associ-ated with a reduced risk of SIDS [85], however, it does not mean that breastfeeding *confers protection* against SIDS, because an infant’s refusal to breastfeed may be a symptom of other SIDS risk factors, like MNC that is known to disrupt breastfeeding [86–88].

The misunderstanding of SIDS is best demonstrated by the popular triple risk hypothesis devised in 1972 by Wedgwood [89], revised in 1994 by Filiano and Kinney [90], and then con-tinuously modified by different authors. This hypothesis pro-poses that factors which increase the risk of sudden death include a critical development period, exogenous stressors, and a vulner-able infant [91]. Filiano and Kinney [90] stated that “an infant will die of SIDS only if he/she possesses all three factors” and emphasized the potential existence of “brain abnormalities.” A later report found enrichment of focal granule cell bilamination in SIDS victims [92] but did not establish causation and due to the choice of controls the commonality of these abnormalities in the general population remained unclear. A comprehensive SIDS investigation sequenced the full exons of 64 genes associ-ated with SIDS in 351 infant and young sudden death decedents [93] found that less than 4% of unexpected deaths were associ-ated with a pathogenic genetic variant. Therefore, the triple risk hypothesis not only fails to explain the main characteristics of SIDS, but its central argument remains unsupported by the ge-netic data.

The allostatic load hypothesis, initially proposed to explain how stress influences the pathogenesis of diseases [94] and later applied to specific disorders (e.g. [95]), proposes that prolonged and repetitive stressful, painful, and traumatic experiences dur-ing the peri-and pre-natal developmental periods lead to the ac-cumulation of allostatic load that may be lethal [5]. Thereby, both hypotheses consider genetic vulnerabilities and external stressors but disagree on the definition of at-risk infants and the sequence of events that leads to SIDS. The allostatic load hy-pothesis considers any infant to be at risk of sudden death in a direct proportion to their genetic vulnerabilities and the cumula-tive stress that they have experienced (a “wear and tear” process) [5] rather than the “intersection” moment of three different risk factors.

Here, we tested some of the predictions of the allostatic load hypothesis [5]. Due to the aforementioned terminological and methodological problems, we sought to focus on the “low hang-ing fruits” – the risk-factors that may explain the characteristics that distinguish SIDS from other deaths: MNC and prematurity. Since these factors are not recorded during autopsies nor can they be linked with hospital records they cannot be studied retroac-tively. We, thereby, carried out an epidemiological study. We found a positive correlation between SIDS mortality and neona-tal circumcision as well as prematurity rates. By large, these phenotypes together were associated with SIDS more than each one separately, suggesting an additive effect, in support of the allostatic load hypothesis [5]. The positive correlations between these phenotype and SIDS are suggestive of the perilous effect that painful and stressful experiences have on infants, particu-larly vulnerable ones.

### 4.1. Evaluating the contribution of MNC toward SIDS

It is well-established that male infants are more susceptible to SIDS than females, but the reason is unclear [96]. The genetic explanations for this phenomenon point at the physiological dif-ferences for cerebral blood flow, neonatal stress, and various in-dices of respiratory function in preterm infants [97] and suggest that preterm males need more respiratory support than females [98]. Other explanations proposed that there exists an X-linked dominant and protective allele (*p* = 1/3) to terminal hypoxia, which leads to a 50% excess in the risk of death for males [99], alternatively there may exist a non-protective X-linked recessive allele (*p* = 2/3) and a protective dominant corresponding X-linked allele (*q* = 1/3) [100]. These explanations assume that the 0.6 average gender bias in US SIDS cases is biologically meaningful. However, the average gender bias in US SIDS cases is inconsistent among US populations (Tables S3). Genetic fac-tors also cannot explain why European countries exhibit differ-ent male biases than US states [101,102].

That SIDS does not have a clear congenital or genetic risk factors seems to preclude the existence of major genetic anoma-lies [103] and highlights the importance of non-genetic factors. When SIDS mortality rates differ between various populations that share the same environment, exploring cultural differences can highlight risk factors for SIDS. For instance, the variabil-ity in SIDS mortality rates (1998–2003) between South Asians (0.2/1000 live births) and White British (0.8/1000) infants who lived in Bradford was explained by the maternal smoking, non-breast feeding, sofa-sharing, and alcohol consumption that were more prevalent in the latter group [104]. In the Netherlands, the higher SIDS mortality rates (1996–2000) in Turkish (0.24/1000) and Moroccan (0.28/1000) infants compared to White Dutch ones (0.16/1000) was associated with customs unique to each group (e.g., side sleeping and the use of pillows). The dangerous combination of bed-sharing and maternal smoking is a common theme identified by several studies that explored the disparities in SIDS mortality rates between different cultures [104,105]. Yet these risk factors cannot explain the high SIDS mortality in US Whites compared to Europeans [46], low SIDS mortality among Ibero-American populations [46,106] compared to US Whites [10], and variable SIDS male-bias observed among US popula-tions.

We argue that the practice of MNC can explain those differ-ences and showed that large proportions of SIDS and SIDS varia-tion between genders in the US can be explained by the MNC but not prematurity rates. Our results suggest that MNC contributes to the high mortality and gender-bias. That the analogous prac-tice of non-therapeutic female genital cutting is illegal in a grow-ing number of countries [107–109] further increases that bias. In addition, females benefit from the protective effect of their sex hormones like estrogen against stressful and painful experiences early in gestation [110–112]. We thereby surmise that the gender variation in SIDS is due to the dual legal-biological protection that females enjoy and that eliminating or postponing MNC may reduce the gender bias but not eradicate it.

Our finding that MNC is associated with SIDS is not sur-prising. Circumcision is associated with intra-operative and postoperative risks, including bleeding, shock, sepsis, circula-tory shock, hemorrhage, pain, and long-term consequences [15– 17,113–116] – all of which contribute toward allostatic load [17– 19] and, thereby, SIDS through various mechanisms [5]. For instance, during circumcision there is an increase in the blood pressure, breathing rate, and heart rate [117,118]. Even with the most advanced techniques, bleeding occurs in over 15% of the cases [119], in which case there is a danger that a lower blood volume would result in low blood pressure and reduced amount of oxygen that reaches the tissues. Reduced blood pressure has been associated with obstructive sleep apnea (OSA), a condition where the walls of the throat relax and narrow during sleep, inter-rupting normal breathing [120,121]. Unsurprisingly, SIDS vic-tims experienced significantly more frequent episodes of OSA [122]. Preterm neonates experience over twice the rate of bleed-ing complications than full-term neonates [123]. MNC-related complications are unavoidable [16–18,123–125]. For instance, in 1949, Gairdner reported [126] that 16 out of 100,000 UK boys under 1-year old died due to circumcision. In tandem with the lack of evidence of a meaningful and relevant health benefits to the infant, several countries chose to opt out of the operation [127].

Until the late 19*^th^* century, Jews were the only group prac-ticing exclusively MNC in Europe [39]. It is, thereby, of interest to ask whether Jewish infants succumb to SIDS at higher rates than other populations? Unfortunately, this question cannot be answered since postmortems are not routinely done in Israel, and SIDS international data do not record religion. An indirect ques-tion would then be, if MNC is a risk factor for SIDS, is there anthropological evidence that Jews acknowledged this associa-tion? Elhaik [5] already showed that MNC was known to be a potentially deadly practice for over a millennium and prompted the splintering of Reform Judaism from Orthodox Judaism in the nineteenth century. Here, we argue that several Jewish customs associated with MNC reflect the footmarks of SIDS, centuries before it was formally defined. Jewish ritualistic circumcision, as practiced today, emerged only during the second century BC [128]. It was also around that time that the myth of the baby-killer Lilith, a beautiful, taloned-foot demoness [129], became prevalent [130]. Originally one of many Mesopotamian demons, Lilith clawed her way through the demonic hierarchy, extend-ing her influence over time until she became Samael’s (Satan) wife around the 13th century [129]. Deceiving Lilith into be-lieving that the newborn was a girl by letting the boy’s hair grow and even dressing him in girls’ clothes during infancy were the most effective means to avoid her harm. This Middle Age tra-dition [131] is still being practiced among Orthodox and even secular Jews who avoid cutting a boys’ hair for the first three years. Other communities adopted a more proactive approach to ward off Lilith and demons during the time of circumcision. The “Night of Watching” ceremony was held on the night pre-ceding circumcision to guard the newborn throughout the night against Lilith [132]. In some ceremonies the guests were pur-posely loud throughout the night to prevent the infant from suc-cumbing to death. Commencing circumcision, Romaniote Jews drew a hand-painted mystical document known as an “Aleph” to protect the child. Overall, these practices are a testament to Jews’ beliefs that 1) sudden death following circumcision was always a non-trivial risk; 2) there exists a major male bias in these otherwise random infant deaths; and 3) sudden death oc-curs at night – all of which are the hallmarks of SIDS. Unfortu-nately, there are limited data of the SIDS mortality rate in Israel due to religious limitations on conducting autopsies [133]. In-terestingly, Israeli health officials reported that, unlike in other countries, Israel saw no reduction in SIDS mortality rate follow-ing the BTS campaign [134].

Contrary to Jews, sufficient data are available for popula-tions whose origin is from the Iberian Peninsula and America. These populations have historically rejected circumcision and, in the US, they continue to resist the procedure despite of their ongoing “Americanization” [40] and the open criticism of US medical institutions on what they consider to be a health risk [135] equal to avoiding vaccination in infants [136]. MNC eva-sion prevailed despite the alleged link between the low MNC and high sexually transmitted diseases (STDs) rates in Hispanic people [135,137]. We found that not only do Hispanic infants succumb less to SIDS but that their SIDS gender bias is closer to one than in non-Hispanic Whites. States with a high Hispanic population have fewer unexplained deaths. This “protective ef-fect,” which extends to non-Hispanic, is difficult to explain with cultural practices that are irrelevant to SIDS infants where 50% of the deaths occur within the first four months of life. We pro-pose that this “Hispanic protective effect” stems from the high proportion (83.5%) of parents who consult with the medical team about the choice of circumcision [138] and the cultural bias of doctors in endorsing the practice [139] as well as the relative ex-posure to members of the Hispanic community who condemn the unwarranted surgery.

### 4.2. Evaluating the contribution of prematurity toward SIDS

The risk of SIDS among preterm infants remained high and unchanged in the US [42] and is inversely associated with ges-tational age [41]. For instance, infants born between 24 to 27 weeks were three times more likely to succumb to SIDS than term infants [41]. The risk factors for SIDS are similar in preterm and term infants, except for parity, which is not asso-ciated with preterm infants [140]. The lowest SIDS mortality rate for preterm infants (*<* 37 weeks) was among Asian/Pacific Islander (1995–1997: 92.8 per 100,000; 2011–2013: 65.2 per 100,000) and Hispanic people (1995–1997: 130.6 per 100,000; 2011–2013: 101.7 per 100,000) [141]. Despite the known male bias in preterm births, we found no association between prema-turity and the gender bias in US SIDS cases, suggesting the ex-istence of stronger factors that determine the gender bias in US populations. Our analyses confirmed that prematurity increases the risk for SIDS and that premature circumcised infants are at a higher risk, in agreement with recent findings indicating that preterm neonates suffer from high rate of bleeding complications following MNC [123], immaturity of their cerebrovascular con-trol in the first year of life [142], and neurodevelopmental com-plications [143,144], which likely contribute toward mortality [41,42]. Our analysis found that circumcision and prematurity are correlated, however it found no interaction between circum-cision and prematurity, i.e., prematurity was not an effect modi-fier and only has an additive effect that in the global model was statistically significant, but not in the US model.

### 4.3. Environmental conditions explain the four main characteristics of SIDS

Our findings explain two out of the four main characteristics of SIDS: male predominance and rarity in Hispanic – both ex-plained by the commonality of MNC. The high mortality rate of SIDS cases during the winter or between the second and forth months after birth can be tenuously explained by the accumu-lation of new stressors, like an increase in respiratory illnesses among household members that are in contact with the infant [145] and the increased sensitivity of infants after their antibody protection weans out [146].

### 4.4. Implications of our findings

Our findings suggest that MNC, the most common pediatric surgery performed on healthy children without a valid medi-cal indication, is a major risk-factor for SIDS. Circumcised in-fants living in a stress-fraught environment, born prematurely, or haveing an existing genetic predisposition to medical condi-tions that may lead to sudden death would be at the highest risk of SIDS. While the risks of preterm births are well-recognized, the debate concerning MNC is polarized between ethical con-cerns [99] and advocacy with respect to contested health benefits [113,147], with few resources devoted to investigating potential long-term risks to infants. Our findings also highlight the impli-cations of US state policy in funding MNC through Medicaid on the risk of SIDS. Although the conclusions of our study should be verified in a cohort study with properly matched infants, some recommendation can be implemented immediately at little cost, such as: eliminating neonatal circumcisions when possible, post-poning non-medical circumcisions to later ages, informing par-ents of the risks in MNC, and applying pain management tech-niques to neonates that experience repetitive pain. MNC data should also be collected and tested in prospective SIDS studies.

### 4.5. Limitations

This study has significant limitations (L1-8), many of which are not due to the study design and are common to all SIDS stud-ies: First, as in all epidemiological studies, correlation is not cau-sation, and causation cannot be inferred from correlation alone. Second, the global MNC rates for two-thirds of the countries were estimated based on the Muslim and Jewish population, with the former known to change their preferences between countries and over time. In the US, per-state MNC rates for some states were only available until 2009. Third, SIDS mortality data were obtained from 15 countries and the unexplained mortality data only from 27 US states, which reduced the power of our analy-ses and may have generated Type I/II errors. Moreover, the SIDS data are not linked with hospital records, which prevents the possibility of retroactive cohort studies. Fourth, pain manage-ment techniques practiced in various countries following MNC could not be accounted for in our study. Fifth, homogeneity of environmental exposure and diagnosis among the SIDS studies has been assumed, but each may be subjected to misclassifica-tion, confounding, and biases. Sixth, we assumed the absence of neonatal female circumcision, which is illegal or uncommon in the studied countries and is rarely practiced at infancy. Sev-enth, the CDC lists SIDS for all autopsied and non-autopsied cases without distinction. In the case of an interracial parent-age, the CDC only reports a single race, usually the one cho-sen by the mother. Finally, countries measure SIDS in different ways, which can contribute discernibly toward the variation in SIDS mortality rates across countries [46]. Changes in the clas-sification of deaths from SIDS to other categories (such as “un-known”) would reduce the SIDS mortality rate and its associa-tion with the phenotypes [148,149]. Unavailability of same-year data for SIDS and the phenotypes may also bias their association.

Some of the above-mentioned limitations were addressed by restricting our analyses to countries that perform autopsies and assembling a secondary dataset of US states. L2) Best-year matched data were used in all the analyses. Although the age of inclusion for SIDS differs across countries, the difference cen-ters on the inclusion of the first week of life, a time when a meager percentage of SIDS deaths occur [10,46]. SIDS mor-tality and the phenotypes’ rates do not change dramatically over time e.g., [10,46,150], thus accepting near year-matched data are likely have a small effect on the results. A major difficulty is to find year-matched MNC and SIDS rates globally. We addressed this problem by deriving the low MNC rates from the proportion of Jewish and Muslims populations who tend to remain constant over short periods of time and showed that halving or doubling their proportions does not change the results. This sensitivity analysis confirmed the robustness of our findings. L4) Stang and Snellman [151] found that most doctors and obstetricians who perform circumcisions avoid using anesthesia due to the ex-tended time the procedure requires (half-hour) and its potentially negative effects [152–154]. L8) We focused on point data col-lected by central sources ([46] or the CDC) and avoid carrying out longitudinal analyses.

Some of the remaining limitations may be addressed in fu-ture cohort studies, but it is likely that other limitations, such as the difficulty in estimating the MNC rates for populations who opt for a private MNC, cannot be addressed, in which case our confidence in the associations depends on their replicability. For that, we showed that the global and US datasets yield similar pat-terns and results in agreement with the biological and historical data and in support of the *allostatic load hypothesis* for SIDS [5].

## 5. Conclusions

SIDS is a diagnosis with a multifactorial underlying etiology. The allostatic load hypothesis [5] explains the main character-istics of SIDS (male predominance, different rates among US groups, mortality rate peaks between 2 and 4 months, and sea-sonal variation) in the prolonged and repetitive stressful, painful, and traumatic stimuli that may begin prenatally, tax neonatal regulatory systems, and increase the risk of SIDS. Our analy-ses support an association between MNC, prematurity, and SIDS and the additive effects of MNC and prematurity toward SIDS. Reducing MNC and preterm rates while mitigating other stres-sors may reduce the mortality rate of unexplained deaths. Our data and code can be used to evaluate associations with other en-vironmental factors. Future cohort studies should consider the existence of MNC, prematurity, genetic vulnerabilities, and life history

## Availability of data and material

All the data and R scripts to generate our figures are available via GitHub.

## Conflict of interest disclosure

E.E consults the DNA Diagnostics Centre (DDC), DNA Consultants, and Mondevices.

**List of abbreviations**
SIDS, sudden infant death syndrome
MNC, male neonatal circumcision
HW, hispanic whites
NHW, non-hispanic white
NHB, non-hispanic blacks
BTS, back to sleep
OSA, obstructive sleep apnea
LR, likelihood ratio
